# COX-2-derived PGE_2_ triggers hyperplastic renin expression and hyperreninemia in aldosterone synthase-deficient mice

**DOI:** 10.1007/s00424-018-2118-z

**Published:** 2018-02-17

**Authors:** Christian Karger, Katharina Machura, André Schneider, Christian Hugo, Vladimir T. Todorov, Armin Kurtz

**Affiliations:** 1 Institute of Physiology, University of Regensburg, Universitätsstraße 31, 93053 Regensburg, Germany; 2 MVZ Dialyse Alter Teichweg, Alter Teichweg 59-61, 22049 Hamburg, Germany; 3 Division of Nephrology, Department of Internal Medicine III, University Hospital Carl Gustav Carus, TU Dresden, Fetscherstraße 74, 01307 Dresden, Germany

**Keywords:** Renin cell hyperplasia, Juxtaglomerular cell, Renin, Prostaglandin

## Abstract

Pharmacological inhibition or genetic loss of function defects of the renin angiotensin aldosterone system (RAAS) causes compensatory renin cell hyperplasia and hyperreninemia. The triggers for the compensatory stimulation of renin synthesis and secretion in this situation may be multimodal. Since cyclooxygenase-2 (COX-2) expression in the macula densa is frequently increased in states of a defective RAAS, we have investigated a potential role of COX-2 and its derived prostaglandins for renin expression and secretion in aldosterone synthase-deficient mice (AS^−/−^) as a model for a genetic defect of the RAAS. In comparison with wild-type mice (WT), AS^−/−^ mice had 9-fold and 30-fold increases of renin mRNA and of plasma renin concentrations (PRC), respectively. Renin immunoreactivity in the kidney cortex of AS^−/−^ mice was 10-fold higher than in WT. Macula densa COX-2 expression was 5-fold increased in AS^−/−^ kidneys relative to WT kidneys. Treatment of AS^−/−^ mice with the COX-2 inhibitor SC-236 for 1 week lowered both renal renin mRNA and PRC by 70%. Hyperplastic renin cells in AS^−/−^ kidneys were found to express the prostaglandin E_2_ receptors EP2 and EP4. Global deletion of EP2 receptors did not alter renin mRNA nor PRC values in AS^−/−^ mice. Renin cell-specific inducible deletion of the EP4 receptor lowered renin mRNA and PRC by 25% in AS^−/−^ mice. Renin cell-specific inducible deletion of the EP4 receptor in combination with global deletion of the EP2 receptor lowered renin mRNA and PRC by 70–75% in AS^−/−^ mice. Lineage tracing of renin-expressing cells revealed that deletion of EP2 and EP4 leads to a preferential downregulation of perivascular renin expression. Our findings suggest that increased macula densa COX-2 activity in AS^−/−^ mice triggers perivascular renin expression and secretion via prostaglandin E_2_.

## Introduction

The synthesis and the secretion of renin in the kidney is the key regulator of the renin angiotensin aldosterone system (RAAS) which controls blood pressure and salt homeostasis of the body [4]. Synthesis and secretion of renin in turn are regulated by the biological efficacy of the RAAS in the sense of a negative feedback [13, 31]. Pharmacological inhibition of RAAS components [16, 40] as well as loss of function mutations of RAAS components [34, 61] therefore lead to increased plasma renin concentrations resulting from an increased number of renin-producing cells [22, 28, 37]. Normally, renin-producing cells are located at the terminal parts of preglomerular arterioles in juxtaglomerular position [1, 62]. In situations of a non-challenged RAAS, renin-producing cells are few in number, but in states of hypotension, sodium deficiency, or RAAS inhibition, additional renin expression is induced in vascular smooth muscle cells and in perivascular and periglomerular interstitial cells [3, 4, 16, 36]. Perivascular and periglomerular renin expression becomes most prominent in genetically caused RAAS defects or salt losses [17, 22, 24, 37, 44, 47, 63]. This ectopic renin expression is not fixed, but instead reacts very sensitively to changes of salt intake [26, 36] indicating continuous regulation of renin synthesis and secretion in perivascular and periglomerular cells. The signals and pathways regulating renin expression in hyperplastic renin cells are not yet clear. They could be causally related to lower blood pressure levels or to sodium deficiency which commonly occur in states of insufficient RAAS activity [56]. They may comprise factors known to regulate renin expression in general such as sympathetic nerves, renal perfusion pressure, nitric oxide, prostaglandins, or others [4, 32]. In particular, prostaglandins have attracted attention as regulators of renin synthesis and secretion [20, 58], mainly because of the rather selective and regulated expression of cyclooxygenase-2 in the macula densa cells of the juxtaglomerular apparatus [19, 21, 23, 30, 38, 45, 66]. In spite of the attractivity of the concept of a macula densa control of renin expression via COX-2 and prostaglandins, this concept has not yet been really proven. Mice lacking COX-2 globally have a lower renin expression and display dysregulations [6, 67], but they are also hypertensive [68] and suffer from severe defects of kidney development [9, 42, 68]. Pharmacological treatment with COX-2 inhibitors frequently lowers renin [18, 41, 64] but these drugs also induce sodium retention [2, 49, 59, 61], what could indirectly also lower renin. Conflicting with the idea that the influence of macula densa COX-2 on renin should be mediated by prostaglandins or prostacyclin may be the observation that apparently microsomal PGE synthase 1 (mPGES-1) which is found in macula densa cells [52] is not required for the strong stimulation of renin synthesis occurring during treatment with loop diuretics [10, 11]. Interestingly, mice lacking PGI2 or EP2 receptors have normal renin [10, 46], whereas mice globally lacking EP4 receptors have reduced renin levels [10, 46, 48], this, however, also in combination with sodium retention.

In view of these findings, we were interested to clearly define a possible role of COX-2 and prostaglandins with their receptors for renin expression by hyperplastic renin cells. As an animal model for strong and reproducible renin cell hyperplasia, we used a mouse lacking aldosterone synthase. In the kidneys of these mice, we have examined the role of COX-2 activity and of prostaglandin receptors for renin synthesis and secretion by hyperplastic renin cells.

## Materials and methods

### Mice

Experiments were performed with inbred 129 SvEv genetic background aldosterone synthase wild-type (WT) and aldosterone synthase-deficient (AS^−/−^) mice at an age of 12 weeks (generously provided by Prof. O. Smithies, University of North Carolina, Chapel Hill, USA) [34]. A global knockout of EP2 receptor was achieved by crossbreeding with mice with targeted disruption of the gene coding for EP2 receptor (EP2^−/−^) [27]. To create a cell-specific EP4 receptor knockout, mice were first crossed with mice harboring loxP flanked EP4 receptor alleles (EP4^fl/fl^) [51]. Mice with EP4 receptor knockout in renin-expressing cells were then generated by crossbreeding with transgenic mice expressing a reverse tetracycline transactivator from the mouse renin gene locus (mRen-rtTAm2) [57] and LC1 mice [53], which express Cre recombinase under the control of a tetracycline response element after doxycycline induction (mRen-rtTAm2/LC1 Cre/EP4^fl/fl^). Lineage tracing of renin-expressing cells was realized by crossing mRen-rtTAm2/LC1 Cre mice with a mouse line containing membrane-Tomato/membrane-GFP (mT/mG) (007676, purchased from Jackson Laboratories, Bar Harbor, USA). This reporter expresses membrane-targeted tdTomato (mT) and the inducible activation of Cre recombinase results in the excision of the tdTomato cassette, which then permits the expression of membrane-targeted enhanced green fluorescent protein (mG) [43]. Animals were kept on standard rodent chow (NaCl 0.6%; Ssniff, Soest, Germany) with free access to tap water. All experiments were conducted according to the National Institutes of Health guidelines for care and use of animals in research. The experiments were approved by the local government.

### Doxycycline treatment for the induction of Cre expression

mRen-rtTAm2/LC1-Cre mice received doxycycline hydrochloride via drinking water ad libitum for 21 days (2 mg doxycycline/ml, 5% sucrose, protected from light, exchanged every 3–4 days) to induce Cre expression, followed by a 10-day period without doxycycline.

### Pharmacological inhibition of COX-2

For pharmacological inhibition of COX-2, mice were given the selective COX-2 inhibitor SC-236 in drinking water (6 mg/L) for 7 consecutive days.

### Determination of renin mRNA expression by real-time PCR

Animals were anesthetized by i.p. injection of ketamine (80 mg/kg body weight; Bela-Pharm, Vechta, Germany) and xylazine (12 mg/kg body weight; Serumwerk, Bernburg, Germany). After ligation of the left renal artery, the kidney was removed, frozen in liquid N_2_, and stored for mRNA analysis. Total RNA was isolated from frozen kidneys as described by Chomczynski and Sacchi [8] and quantified using a photometer. The cDNA was synthesized by MMLV reverse transcriptase (Superscript; Invitrogen, Carlsbad, USA). For quantification of renin mRNA expression, real-time PCR was performed using a Light Cycler 480 Instrument (Roche Diagnostics Corp., Mannheim, Germany) and the Light Cycler SYBR Green I Master Kit (Roche Diagnostics Corp., Mannheim, Germany) and glyceraldehyde-3-phosphate dehydrogenase (GAPDH) or ribosomal protein L32 (RPL32) as a control. For amplification of cDNAs, the following primers were used: COX-2 sense 5′-agccatttccttctctcctg-3′; COX-2 antisense 5′-acaacaactccatcctcctg-3′; GAPDH sense 5′-caccagggctgccatttgca-3′; GAPDH antisense 5′-gctccacccttcaagtgg-3′; renin sense 5′-atgaagggggtgtctgtggggtc-3′; renin antisense 5′-atgcggggagggtgggcacctg-3′; RPL32 sense 5′-ttaagcgaaactggcggaaac-3′; RPL32 antisense 5′-ttgttgctcccataaccgatg-3′.

### Determination of plasma renin concentration

For the determination of plasma renin concentration, blood samples taken from the tail vein were centrifuged and the plasma was incubated with plasma from bilaterally nephrectomized male rats as renin substrate for 90 min at 37 °C. The produced angiotensin I (ng/ml·h^−1^) was determined by ELISA (Angiotensin I Plasma Renin Activity ELISA; IBL international, Hamburg, Germany).

### Immunohistochemistry

As described previously [50], kidneys were perfusion-fixed with 3% paraformaldehyde, dehydrated, and embedded in paraffin. Immunolabeling was performed on 5-μm paraffin sections. After blocking with 10% horse serum and 1% BSA in PBS, sections were incubated with goat anti-mouse renin (1:400; Cat. No. AF4277; R&D Systems, Minneapolis, USA) and α-smooth muscle actin (SMA) (1:600; Cat. No. ab7817; Abcam, Cambridge, UK), GFP (1:600; Cat. No. ab13970; Abcam, Cambridge, UK) antibodies overnight at 4 °C, followed by incubation with Cy5-, Cy2-, and TRITC-labeled secondary antibodies (1:400; Dianova, Hamburg, Germany). For COX-2 staining (1:200; Cat. No. 160126; Cayman, Ann Arbor, USA), sections were incubated in Tris/EDTA (heated to 95 °C in a water bath) for 45 min before blocking with 5% milk powder/PBS. Slices were mounted with Dako Cytomation Glycergel mounting medium (Agilent, Santa Clara, USA) and viewed with an Axiovert microscope (Zeiss, Oberkochen, Germany). As a technical note, it should be added that the chosen fixation protocols and embedding procedures destroy native GFP and tomato fluorescence. For quantification of areas immunoreactive for renin, the obtained images were subsequently analyzed using ImageJ software (National Institutes of Health, Bethesda, USA). Assumed background areas were excluded by blinded manual thresholding and the area of fluorescence was compared to the total kidney cortex area.

### In situ hybridization

Localization of EP2 receptor, EP4 receptor, and PDGF-receptor β mRNA synthesis was studied by a novel in situ hybridization technology using the RNAscope® 2.5 HD Duplex Assay (Advanced Cell Diagnostics, ACD, Hayward, USA), according to the manufacturer’s instructions [65]. Kidneys were perfusion-fixed with 10% neutral buffered formalin solution, dehydrated in a graduated ethanol series, and embedded in paraffin. Five-micrometer sections were treated with peroxidase blocker before boiling at 90–100 °C in a pretreatment solution for 15 min, and protease was then applied for 30 min at 40 °C. Target probes (designed by ACD: EP2 receptor, Cat. No. 456481; EP4 receptor, Cat. No. 441461; PDGF-receptor β, Cat. No. 411381-C2) were hybridized for 2 h at 40 °C, followed by a series of signal amplification and washing steps. All hybridizations at 40 °C were performed in a HybEZ Hybridization System (ACD). Hybridization signals were detected by chromogenic precipitate development. RNA staining signal was identified as red and blue punctate dots. Following the RNAscope assay, samples were counterstained for 90 s with 50% Gill’s hematoxylin diluted in dH_2_O. Slices were mounted with VectaMount mounting medium (Vector Laboratories, Burlingame, USA) and were viewed with an Axiovert microscope (Zeiss, Oberkochen, Germany).

### Statistics

All data are presented as the mean ± SEM. Differences between groups were analyzed by *t* test or ANOVA and Bonferroni’s adjustment. *P* values less than 0.05 were considered statistically significant. Prism 5.0 software (GraphPad, San Diego, USA) was used for all calculations.

## Results

**Fig. 1:**
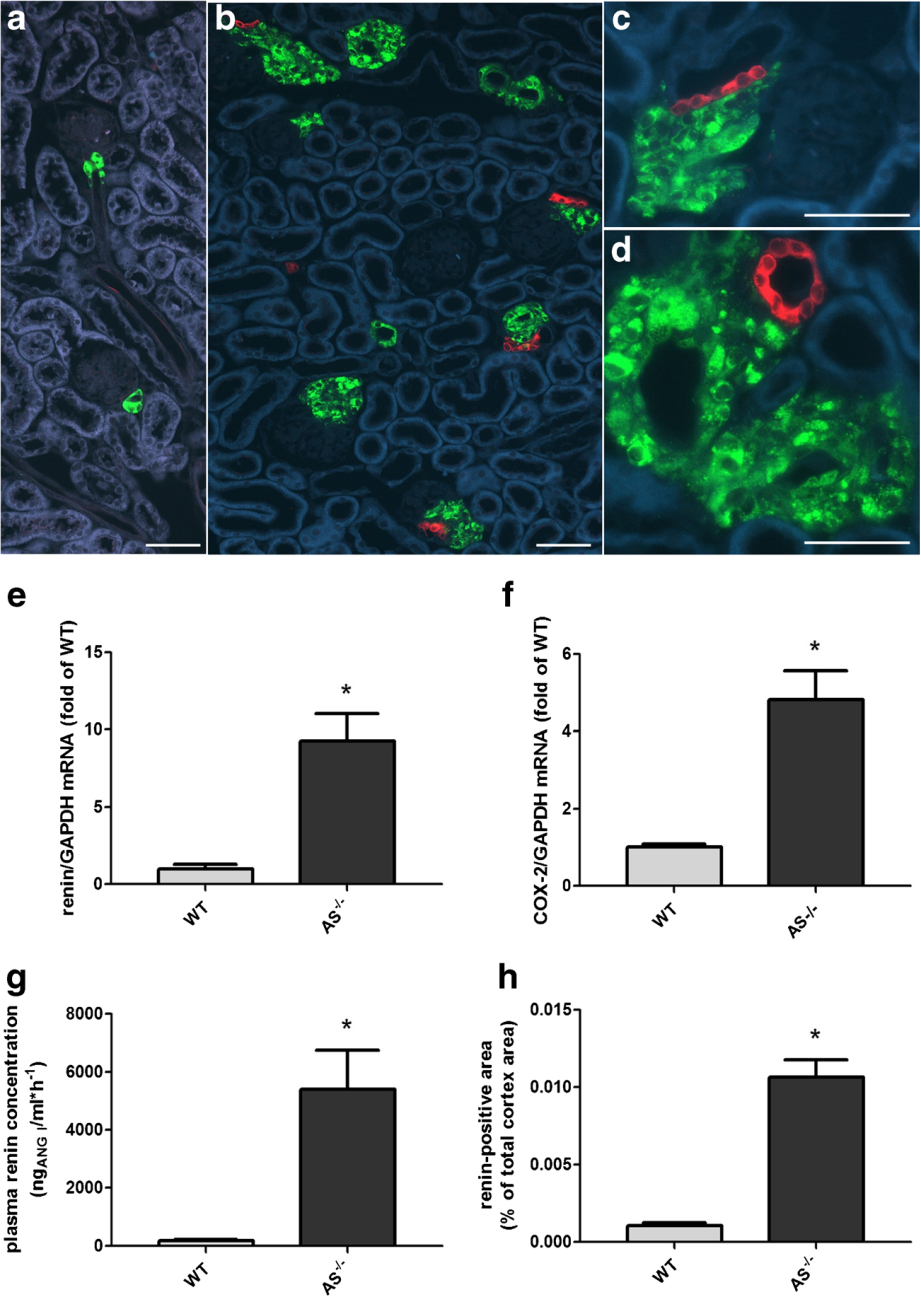
(A–D) Immunohistochemistry of COX-2 (red) and renin (green) in kidney sections of a wildtype mouse (A) and a mouse lacking aldosterone synthase (B–D)*.* Renin cells in wild-type kidneys were small in number and located in the classical juxtaglomerular position within the media layer of the afferent arteriole. In contrast, kidneys of the AS^−/−^ mouse showed a strong increase in the number of renin-positive cells surrounding afferent arterioles. In direct proximity to the renin producing cells, tubular cells of the AS^−/−^ mice showed a strong expression of COX-2, whereas no COX-2 staining was found in the cortex of wild-type mice. Bars indicate 50 μm. (E–H) Renin and COX-2 expression is increased in kidneys of AS^−/−^ mice. E and F show renin mRNA abundance and COX-2 mRNA abundance in kidneys of WT and AS^−/−^ mice. G shows plasma renin concentration in WT and AS^−/−^ mice. H shows quantitative analysis of renin immunofluorescence in kidney cortices of WT and AS^−/−^ mice. Data are means ± SEM of 6–18 mice of each group. Asterisks indicate *p* < 0.05

Histological analysis of kidneys of mice lacking aldosterone synthase showed a clear hyperplasia of renin-producing cells (Fig. 1). Renin-positive cells were not only located in the classical juxtaglomerular region but also found within the media of more distal parts of the afferent arteriole and in cuff-like multilayers surrounding preglomerular vessel walls. In close proximity to the hyperplastic renin cells, we found a strong signal of COX-2 in tubular macula densa cells where the tubular system comes into direct contact with the juxtaglomerular area (Fig. 1). In wild-type kidneys, no COX-2 signal was detectable (Fig. 1).

The higher number of renin-producing cells in AS^−/−^ mice was reflected by a 9-fold increase in renal renin mRNA abundance and a 30-fold increase in plasma renin concentration in comparison to WT controls (Fig. 1). Quantification of renin immunofluorescence showed that the total area of renin-immunoreactive cells was 10-fold higher in the cortex of AS^−/−^ mice than in WT (Fig. 1). COX-2 mRNA abundance in the cortex of AS^−/−^ kidneys was 5-fold higher than in WT (Fig. 1).

To investigate a possible effect of COX-2 on renin expression in AS^−/−^ mice, we inhibited COX-2 activity by the use of the selective COX-2 inhibitor SC-236 [29]. Similar to our previous results with rats, we found that SC-236 lowered urinary PGE_2_ concentrations by about 60% [39].

**Fig. 2:**
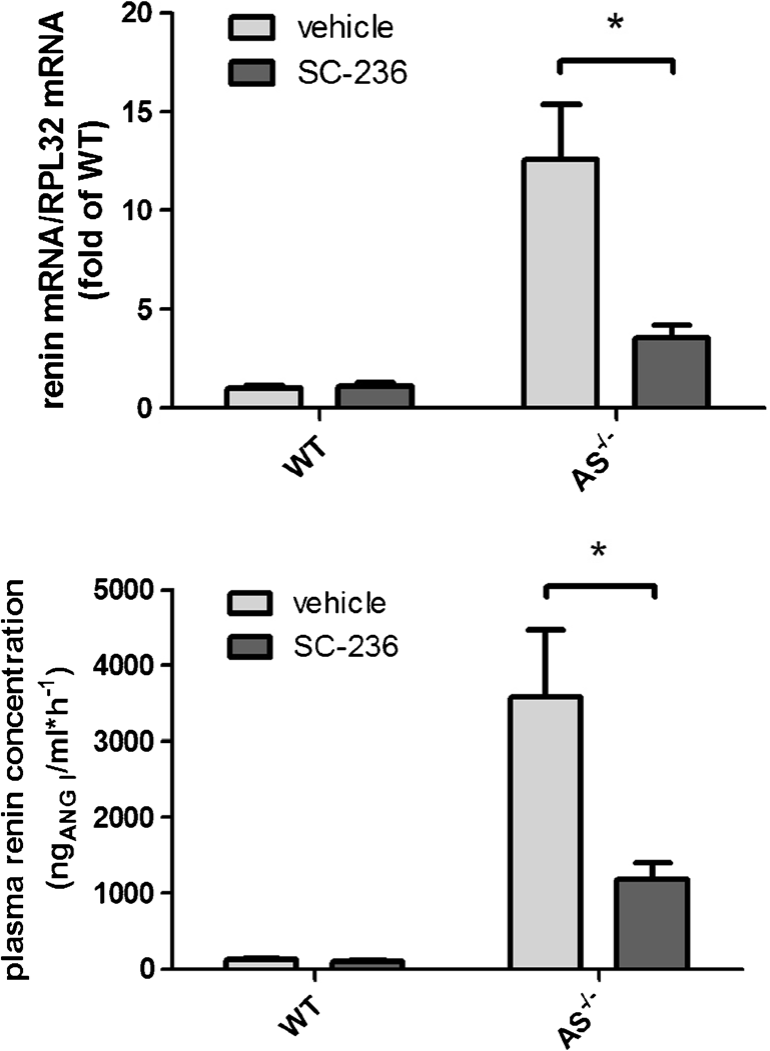
Renin mRNA and plasma renin concentration are sensitive to COX-2 inhibition in AS^−/−^ mice. Kidney renin mRNA abundance (upper panel) and plasma renin concentration (lower panel) in AS^−/−^ mice and in wild-type controls were measured under basal conditions and after 1 week of SC-236 treatment. Data are means ± SEM of 6 mice of each genotype. Asterisks indicate *p* < 0.05

Treatment with SC-236 (1 mg/(d·kg)) for 1 week led to a 70% decrease in renin mRNA abundance in the kidneys of mice lacking aldosterone synthase. In accordance, plasma renin concentration in AS^−/−^ mice was also lowered by 70% after treatment with SC-236 (Fig. 2). In WT mice, selective COX-2 inhibition had no effect on renin mRNA levels and plasma renin concentrations (Fig. 2).

To examine if the effect of COX-2 on renin expression was related to PGE_2_ formation, we next looked for the existence of PGE_2_ receptors in hyperplastic renin cells. We focused on receptors that are able to activate adenylyl cyclase, because cAMP is considered as the major trigger of renin gene transcription and renin secretion [4] in hyperplastic renin cells.

**Fig. 3:**
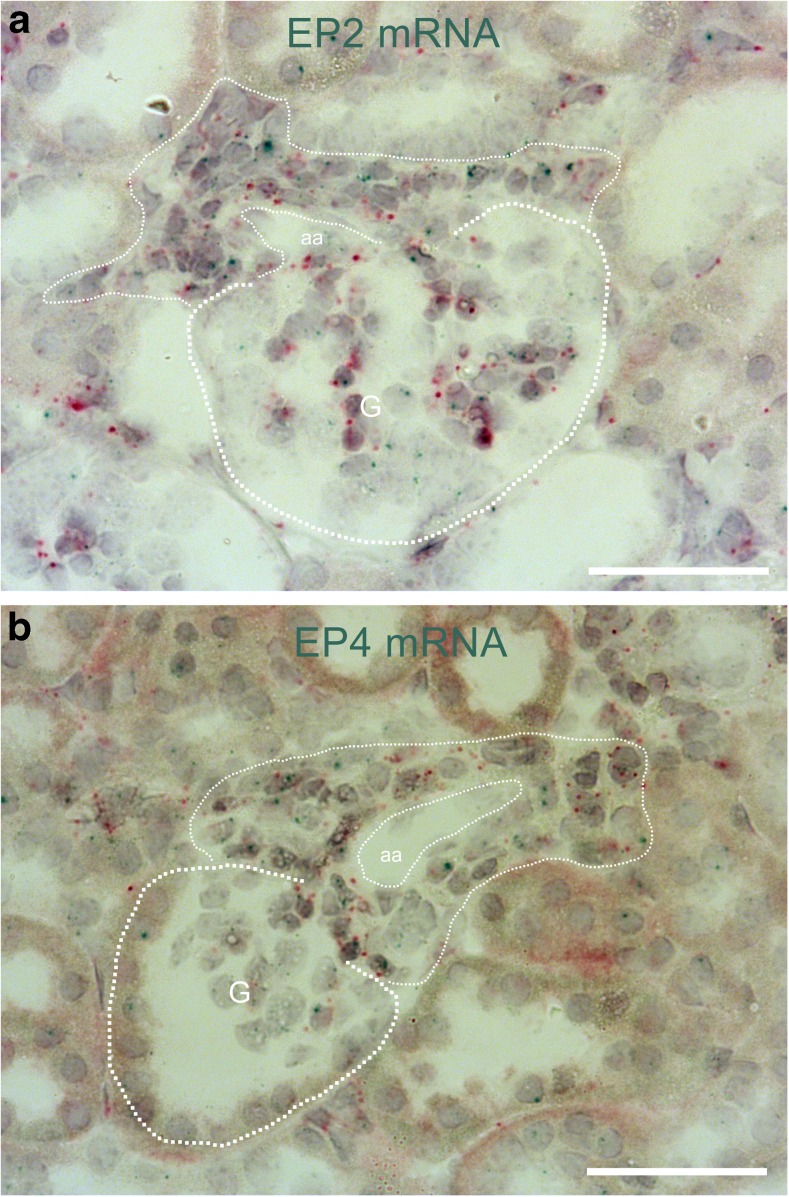
Hyperplastic renin cells in AS^−/−^ mice express EP2 and EP4 receptors. In situ hybridization with the RNAscope method showed clear expression of both EP2 receptor mRNA (A; blue dots) and EP4 receptor mRNA (B; blue dots) in hyperplastic renin cells of AS^−/−^ mice. Hyperplastic renin cells (thin dotted line) were identified as PDGFR-β mRNA-expressing (red dots) granular cells, surrounding afferent arterioles (aa). G and thick dotted lines indicate glomeruli. Bars indicate 50 μm

**Fig. 4:**
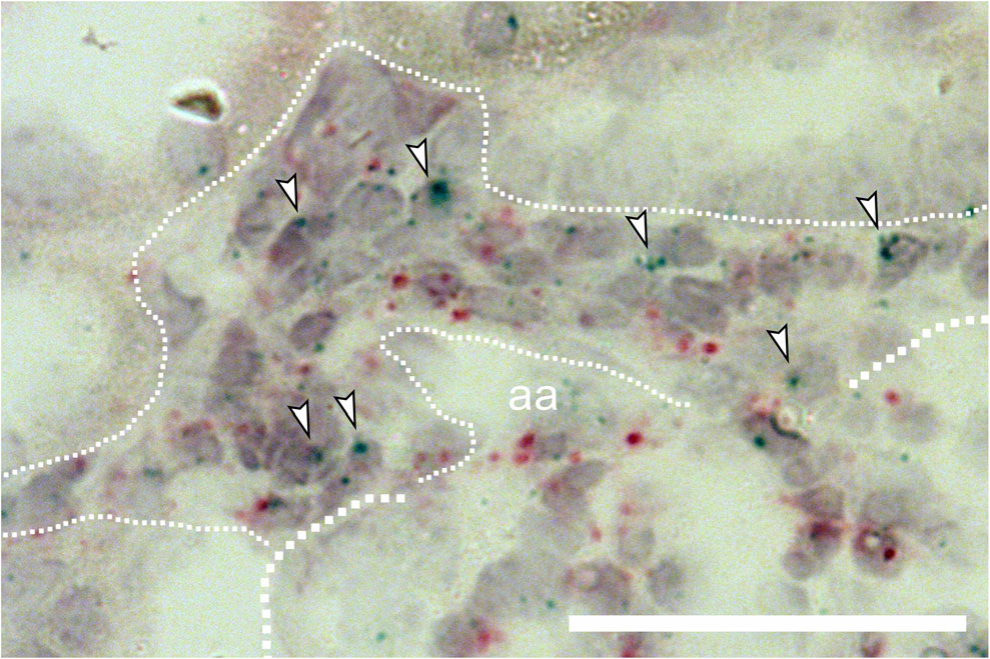
Enlarged view of EP2 receptor mRNA in situ hybridization (blue dots) in hyperplastic renin cells of AS^−/−^ mice. Arrowheads highlight selected EP2 receptor mRNA expression signals. Thick dotted line indicates a glomerulus. Thin dotted line indicates hyperplastic renin cells. aa, afferent arteriole; red dots, PDGFR-β mRNA expression. Bar indicates 50 μm

**Fig. 5:**
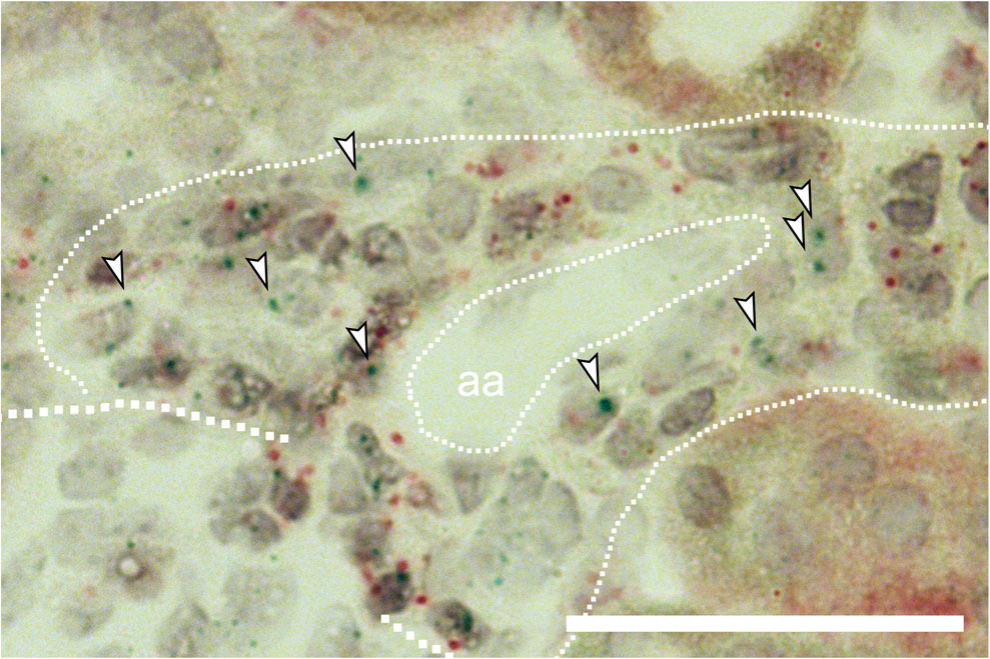
Enlarged view of EP4 receptor mRNA in situ hybridization (blue dots) in hyperplastic renin cells of AS^−/−^ mice. Arrowheads highlight selected EP4 receptor mRNA expression signals. Thick dotted line indicates a glomerulus. Thin dotted line indicates hyperplastic renin cells. aa, afferent arteriole; red dots, PDGFR-β mRNA expression. Bar indicates 50 μm

By the use of mRNA in situ hybridization, we found that the hyperplastic renin cells of aldosterone synthase-deficient mice distinctly expressed the cAMP stimulatory prostanoid receptors EP2 and EP4 (Figs. 3, 4, 5).

**Fig. 6:**
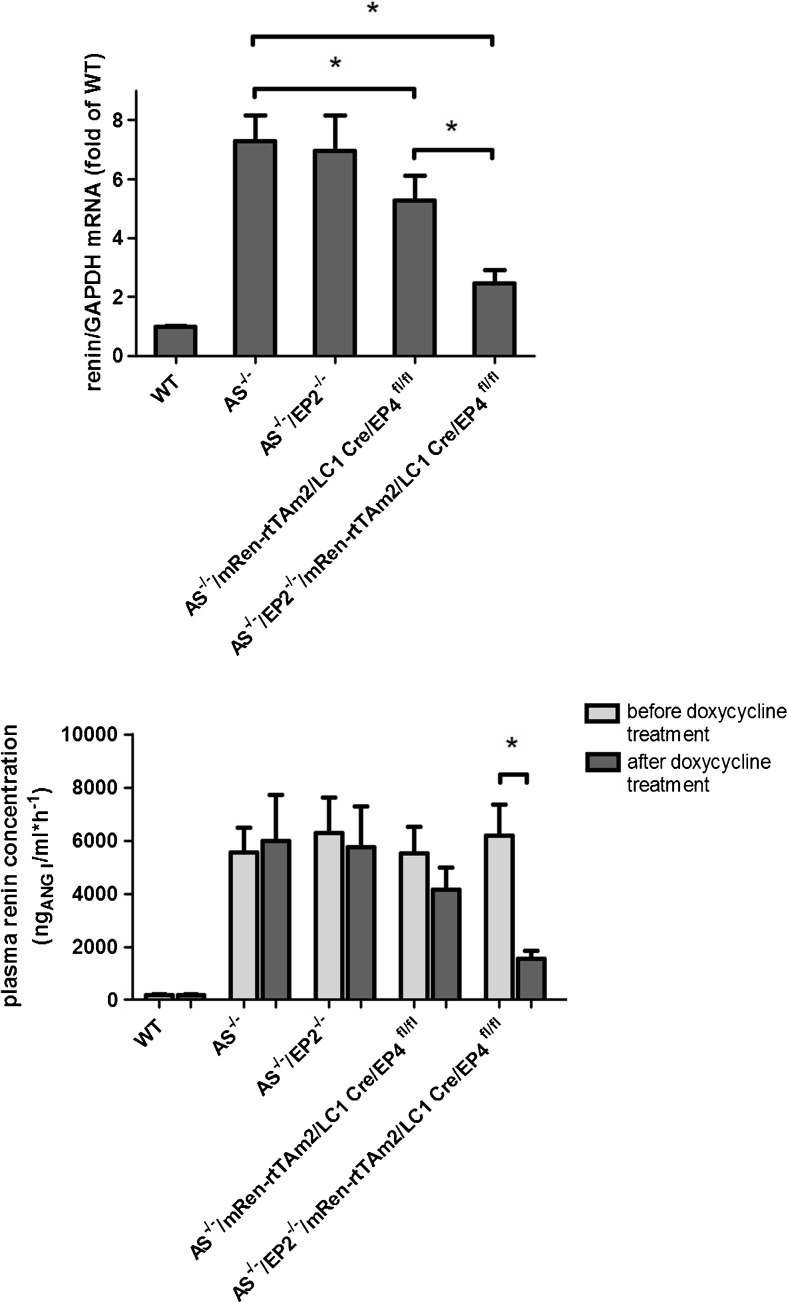
Deletion of EP2/EP4 receptors in renin cells lowers renin mRNA and plasma renin concentration. Kidney renin mRNA abundance (upper panel) and plasma renin concentration (lower panel) in wild-type, AS^−/−^, AS^−/−^/EP2^−/−^, AS^−/−^/mRen-rtTAm2/LC1 Cre/EP4^fl/fl^, and AS^−/−^/EP2^−/−^/mRen-rtTAm2/LC1 Cre/EP4^fl/fl^ mice. Renin mRNA abundance was measured 10 days after 21 days of doxycycline treatment. Plasma renin concentration was measured before and 10 days after 21 days of doxycycline treatment. Data are means ± SEM of 5–20 mice of each genotype. Asterisks indicate *p* < 0.05

**Fig. 7:**
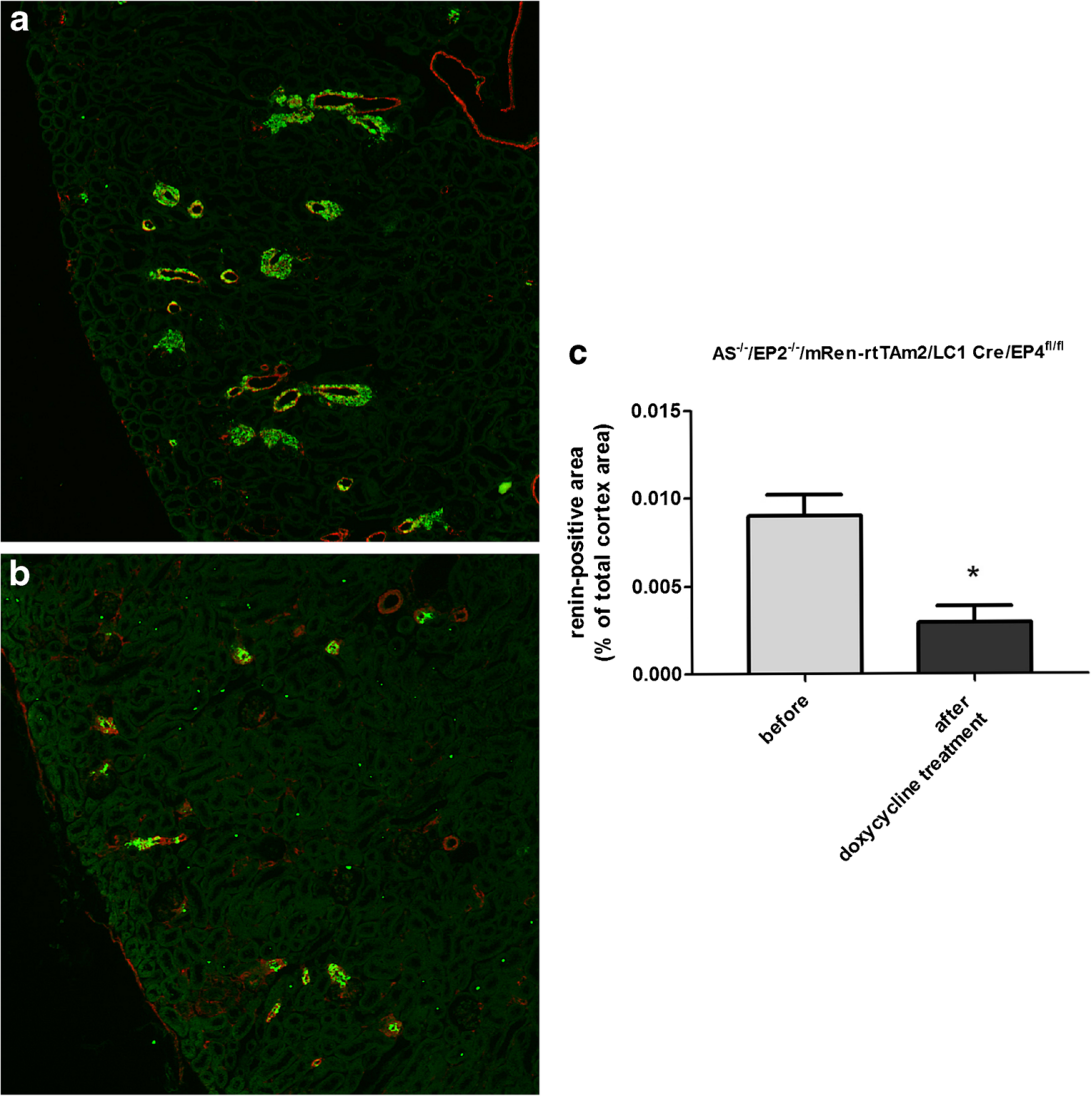
Deletion of EP2/EP4 receptors in renin cells reduces renin immunoreactivity in the cortex of AS^−/−^ mice. Immunostaining (A, B; Renin, green; α-SMA, red) and quantitative analysis of renin immunofluorescence (C) showed a clear decrease in renin-positive areas in the cortex of AS^−/−^ mice after deletion of EP2 and EP4 in renin cells. Kidneys of AS^−/−^/EP2^−/−^/mRen-rtTAm2/LC1 Cre/EP4^fl/fl^ were analyzed before and 10 days after 21 days of doxycycline treatment. Data are means ± SEM of 4 mice of each group. Asterisks indicate *p* < 0.05. Bars indicate 100 μm

A global deletion of the EP2 receptor in aldosterone synthase-deficient mice (AS^−/−^/EP2^−/−^) had no influence on renin mRNA and plasma renin concentrations (Fig. 6). The deletion of the EP4 receptor specifically in renin-expressing cells after doxycycline-dependent induction (AS^−/−^/mRen-rtTAm2/LC1 Cre/EP4^fl/fl^) led to a moderate decrease in renin mRNA by around 25% whereas the decline in plasma renin concentrations did not reach statistical significance (Fig. 6). However, renin cell-specific deletion of the EP4 receptor in the absence of the EP2 receptor (AS^−/−^/EP2^−/−^/mRen-rtTAm2/LC1 Cre/EP4^fl/fl^) had a strong effect on renin mRNA levels and plasma renin concentrations in AS^−/−^ mice. Renin mRNA was decreased to around 33% and plasma renin concentrations were lowered to 25% of the initial value after induced renin cell-specific deletion of the EP4 receptor (Fig. 6). Histological analysis of the kidneys of these mice revealed a clear reduction in the number of renin-positive cells after EP2/EP4 deletion. Renin-positive area in the cortex of AS^−/−^/EP2^−/−^/mRen-rtTAm2/LC1 Cre/EP4^fl/fl^ after doxycycline induction was one third of the size before induction (Fig. 7).

To follow the fate of hyperplastic renin cells in AS^−/−^ mice after the combined deletion of EP2 and EP4 receptor, we traced the lineage of renin-producing cells by the use of a doxycycline inducible Cre-dependent double fluorescent reporter (mT/mG). With this reporter, mice expressed membrane-targeted Tomato (mT) prior to Cre-mediated excision and membrane-targeted GFP (mG) after excision.

**Fig. 8:**
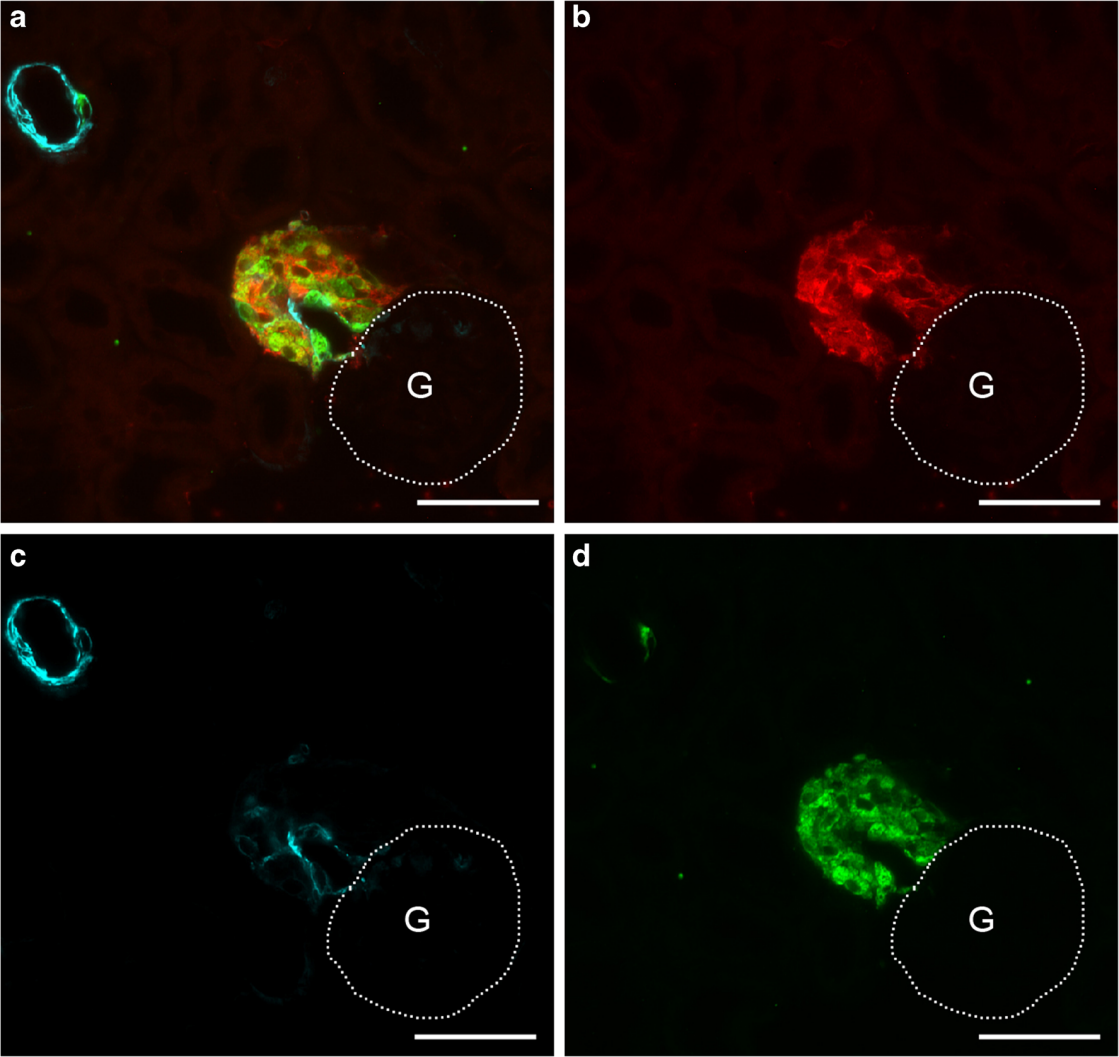
Renin cell lineage labeling in AS^−/−^/mRen-rtTAm2/LC1 Cre/mT/mG mice. After doxycycline induction, GFP-expression was irreversibly switched on in all vascular and perivascular renin cells. A shows a combined image of GFP (red), α-SMA (cyan), and renin (green) immunoreactivity. B–D shows immunoreactivity for GFP, α-SMA, and renin alone. G and dotted lines indicate glomeruli. Bars indicate 50 μm

**Fig. 9:**
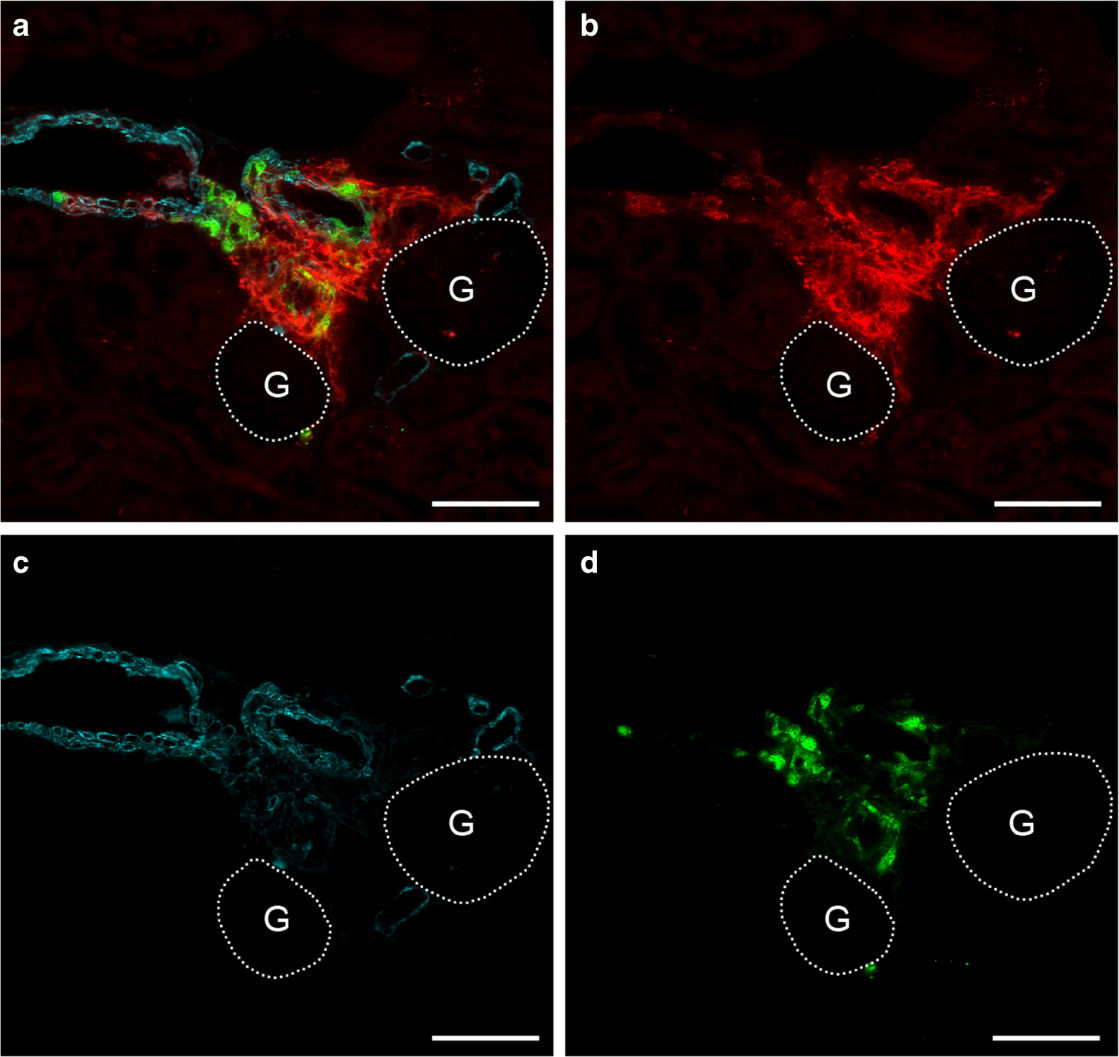
Renin cell lineage tracing reveals perivascular downregulation of renin expression after EP2 and EP4 deletion in AS^−/−^ mice. Deletion of EP2 and EP4 in AS^−/−^/EP2^−/−^/mRen-rtTAm2/LC1 Cre/EP4^fl/fl^/mT/mG mice results in a clear reduction of the renin signal and a persistence of large areas of perivascular GFP-expressing renin-negative cells 10 days after doxycycline induction. The decrease in renin expression was mainly seen in the perivascular renin cells while in vascular renin cells, expression of renin was not affected. A shows a combined image of GFP (red), α-SMA (cyan), and renin (green) immunoreactivity. B–D shows immunoreactivity for GFP, α-SMA, and renin alone. G and dotted lines indicate glomeruli. Bars indicate 50 μm

After the administration of doxycycline, GFP expression was irreversibly switched on in all renin-expressing cells of AS^−/−^/mRen-rtTAm2/LC1 Cre/mT/mG mice (Fig. 8). Ten days after doxycycline induction, the combined deletion of EP2 and EP4 receptors in renin-producing cells (AS^−/−^/EP2^−/−^/mRen-rtTAm2/LC1 Cre/EP4^fl/fl^/mT/mG) resulted in a clearly reduced number of renin-positive cells. Remaining renin cells were found mainly within the vessel walls of afferent arterioles whereas perivascular renin was clearly reduced. However, the persistence of perivascular GFP-expressing renin-negative cells indicates that the reduction in perivascular renin was not due to a disappearance of perivascular cells (Fig. 9).

## Discussion

In accordance with previous reports [34, 37], we found strong perivascular and periglomerular renin cell hyperplasia in mice lacking aldosterone synthase. We could also confirm an enhanced expression of cyclooxygenase-2 in the macula densa cells of aldosterone synthase-deficient mice [34, 37]. An impact of COX-2 for renin expression and renin secretion in these mice was indicated by the effect of the COX-2 blocking drug SC-236 which substantially lowered the strongly enhanced kidney renin mRNA levels and plasma renin concentrations in aldosterone synthase-deficient mice. An effective renin suppression by COX-2 inhibition has already been described for adrenalectomized rats [60]. These observations are in line with a more general renin inhibitory effect of COX-2 blockers, in particular in situations of a stimulated renin synthesis and secretion [18, 25, 64]. Since the cyclic AMP signaling pathway is the yet best characterized trigger for renin gene transcription and for renin secretion [33, 35, 54], we next looked for the existence of cAMP stimulatory prostanoid receptors in renin-expressing cells. Apart from prostacyclin receptors, the PGE_2_ EP2 and EP4 receptors are the yet best established activators of adenylyl cyclase activity in target cells [12, 55]. Indeed we obtained clear evidence for the expression of EP2 and of EP4 receptors in renin cells, particularly in hyperplastic renin cells. The effect of deletion of these receptors on renin gene expression and on plasma renin concentration was analyzed in AS^−/−^ mice with a global deletion of the EP2 receptor (AS^−/−^/EP2^−/−^), an inducible renin cell-specific deletion of the EP4 receptor (AS^−/−^/mRen-rtTAm2/LC1 Cre/EP4^fl/fl^) or a combined deletion of both EP2 and EP4 receptors (AS^−/−^/EP2^−/−^/mRen-rtTAm2/LC1 Cre/EP4^fl/fl^). Global deletion of the EP2 receptor had no effect on renin gene expression and renin secretion in aldosterone synthase-deficient mice. A similar lacking effect of EP2 receptor deletion on renin synthesis and secretion has already been reported in mice in which renin expression had been stimulated either by inhibition of the angiotensin I-converting enzyme or by loop diuretics [7, 10, 46]. Inducible deletion of the EP4 receptor specifically in renin cells led to a moderate decrease of renin mRNA and of plasma renin concentration. In mice with global deletion of the EP4 receptor, an attenuated stimulation of renin by treatment with loop diuretics or with low-salt diet has already been reported [10, 46, 48]. Mice with global deletion of EP4 receptors, however, show sodium retention [46, 48], which may enhance a direct effect resulting from the lack of EP4 receptors on renin cells. Inducible deletion of EP4 receptors in renin cells lacking also EP2 receptors now produced a prominent attenuation of renin gene expression and of plasma renin concentration suggesting that EP2 and EP4 receptors are essential for renin expression and that EP2 receptor may compensate, if the EP4 receptor is lacking. Renin cell lineage tracing revealed that deletion of EP2/EP4 receptors silences renin expression mainly in perivascular cells. Our findings also suggest that a stop of renin expression by interrupting PGE_2_ signaling on renin cells does not lead to cell death and disappearance. This finding supports our previous assumption of a reversible switch “on and off” of renin expression in hyperplastic perivascular renin cells [26, 36].

Altogether, our data strongly suggest that PGE_2_ is required to maintain renin expression in hyperplastic renin cells. Via EP2 and EP4 receptors, PGE_2_ likely activates the cyclic AMP pathway which is critical for renin gene transcription. It should be noted that EP4 but not EP2 receptors can activate the PI3K/PKB [14, 15], which is relevant for cell differentiation [5]. If this additional effect of EP4 receptors is required for renin cell hyperplasia, it might explain why EP2 receptor deletion alone exerted no effect on renin gene expression. PGE_2_ required for hyperplastic renin expression likely results from COX-2 activity. From the parallel changes of perivascular renin and of macula densa COX-2 expression, it is intriguing to speculate that the macula densa is the source of PGE_2_ triggering hyperplastic renin expression.
